# Spinal reflexive movement follows general tau theory

**DOI:** 10.1186/s12868-021-00626-3

**Published:** 2021-04-01

**Authors:** Mehrdad Bahadori, Paola Cesari, Cathy Craig, Mehran Emadi Andani

**Affiliations:** 1 Department of Neuroscience, Biomedicine, and Movement Sciences, University of Verona, Via Casorati 43, 37131 Verona, Italy; 2 School of Psychology, Ulster University, Coleraine, Northern Ireland; 3 Department of Biomedical Engineering, Faculty of Engineering, University of Isfahan, Isfahan, Iran

**Keywords:** Tau theory, Involuntary movements, Gravitational field, Movement planning, Patellar reflex

## Abstract

**Background:**

Tau theory explains how both intrinsically and perceptually guided movements are controlled by the brain. According to general tau theory, voluntary, self-paced human movements are controlled by coupling the tau of the movement (i.e., the rate of closure of the movement gap at its current closure rate) onto an intrinsically generated tau-guide (Lee in Ecol Psychol 10:221–250, 1998). To date there are no studies that have looked at involuntary movements, which are directly guided by innate patterns of neural energy generated at the level of the spinal cord or brain, and that can be explained by general tau theory. This study examines the guidance of an involuntary movement generated by the Patellar reflex in presence of a minimized gravitational field.

**Results:**

The results showed that the Patellar reflexive movement is strongly coupled to an intrinsic tau-guide particularly when the limb is not moving in the direction of gravity.

**Conclusion:**

These results suggest that the same principles of control underpin both voluntary and involuntary movements irrespective of whether they are generated in the brain or the spinal cord. Secondly, given that movements like the patellar reflex are visible from infancy, one might conclude that tau-guidance is an innate form of motor control, or neural blueprint, that has evolved over time.

## Background

Movement, and the way it is guided or controlled by the central nervous system, has always been of great interest to researchers. To date, human movement has been studied from both perceptual and motor perspectives resulting in different theories of how it is controlled [1, 2]. General tau theory is one such theory that suggests that the patterning of perceptual information, can be picked up and used by the central nervous system to temporally guide the closure of action gaps. This theory was first proposed by David N. Lee in 1976 who looked at how timing information directly detected through changing patterns in the optic array can be picked up and used to prospectively control any subsequent movement. Examples have included braking when driving a car or moving to intercept a ball. The theory later evolved to explain how other patterns of temporal information picked up through other sensory arrays (extrinsic control), but also generated by the central nervous system (intrinsic control), can prospectively control other forms of biological movement [3–6]. The central tenet of this theory is that the taus of different types of action gap are closely coupled together [4]. An action gap is defined as the changing gap between a current state and the goal state. Tau ($\tau$), an informational variable that blends space and time, specifies the time to closure of an action gap (e.g., *x*) at its current closure rate ($\tau_{x} = x/\dot{x}$; *x* is the magnitude of the action gap and x dot signifies its rate of change at each moment in time). Action gaps can be perceived through the patterning of information picked up through the senses (extrinsic tau) [7] or generated intrinsically by the CNS (intrinsic tau-guide).

For instance, when catching a ball, the ball’s arrival position and time to arrival (tau of the ball_hand gap), is specified by the patterning of information generated by the changing optic array and picked up through the eyes [8]. On the other hand, when intercepting a virtually moving sound source, the position and arrival time of the sound is specified through the patterning of auditory information (tau) and is picked up by the auditory system [9]. According to general tau theory, successfully intercepting a target requires that the tau of the hand-target action gap ($\tau_{y}$) is tightly coupled to the tau of the hand-arrival position action gap ($\tau_{x}$) ($\tau_{y} = K\tau_{x}$) to ensure they both close at the same time. The coupling coefficient *k* (a constant) describes how the gaps close together [4].

For self-paced movements such as reaching for a glass of water, or putting a ball in golf, there is no dynamic external information that can be picked up by the senses to guide the temporal control of these movements. In these examples, as with any purposeful movement, it is critically important that the movements are controlled prospectively. In these examples, where there is no extrinsic (sensory) information to assist with the timing of the movement, it is hypothesized that an intrinsic pattern of information generated by the nervous system guides the control of the movement [7]. The taug equation that specifies the patterning of neural energy needed to guide these movements depends on the duration of the movement and is defined as: $\tau_{g} = 0.5\left( {t - T^{2} /t} \right)$ [4, 7]. $T$ is the duration of movement and *t* is time as the movement unfolds (from movement initiation to the end of the movement). Research has shown that in the case of moving one’s hand to one’s mouth, putting a ball in golf (both self-paced actions) and maintaining dynamic balance during gait initiation that the movements are tightly coupled to an intrinsic tau-guide [10–12]. Indeed, other research has shown that different levels of skill are manifested through different coupling coefficients, with highly skilled participants having higher coupling coefficients compared to the non-skilled [13]. In an attempt to understand the neural mechanisms underpinning this form of intrinsic information/movement coupling, other researchers have shown evidence for taug coupling inside the (infant) brain [14, 15].

However, up until now, general tau theory has only looked at voluntary movements which require temporal control and prospective guidance [8, 9, 11, 12]. To make a voluntary movement, signals generated in the primary motor cortex are first carried by the corticospinal tract to a particular spinal cord segment, before exiting the spinal cord to reach the specific muscles required to produce that particular movement [16].

Reflexive movements, on the other hand, are generated in an involuntary manner with some reflexes being present from birth [17]. In the case of spinal reflexes, the brain receives the activation message from the onset of the movement [18]. Reflexes are categorized into three types: (1) spinal reflexes e.g., patellar reflex, (2) cortex reflexes e.g., dermal reflexes, and (3) the reflexes of the posterior parts of the brain responsible for complex reflexes such as swallowing [19]. Considering the number of neurons engaged in the reflex, reflexes are either monosynaptic or polysynaptic.

Since no study has looked at the control of involuntary movements, the main aim of this study was to see if general tau theory could explain the temporal control of reflexive type movements. We chose to examine the Patellar reflex, one of the primary reflexes that is present from early infancy [20, 21]. This reflex is classed as a monosynaptic and tendinous reflex generated by direct command from the spinal cord [22].

**Fig. 1:**
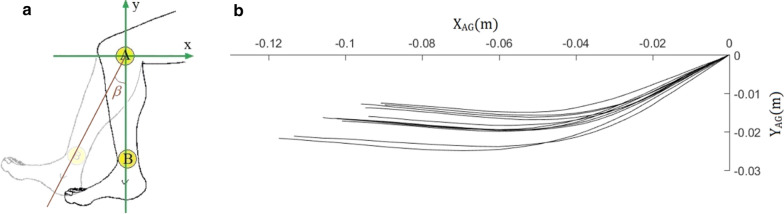
**a** A and B markers placed on the knee and ankle (yellow circles) and used to calculate the angle *β* (i.e., the angle of the leg with respect to the vertical axis). **b** Ten representative ankle movement action gaps from one individual participant in vertical condition

Before continuing, it is important to understand the sequence of events that unfold when the Patellar reflex is initiated and how voluntary control kicks in to bring the leg back to a resting position. Firstly, the quadriceps tendon is suddenly and briefly stretched by the hammer blow. This briefly stretches the quadriceps muscle, which in turn briefly stretches the spindles in the quadriceps, thereby briefly increasing their tension. This fleeting increase in tension causes the quadriceps muscle to briefly contract to reduce the tension in the spindles. The contraction of the quadriceps muscle starts as the leg moves on its upward course. As the leg moves upward, the length of the quadriceps muscle starts to decrease at a normal (non-jerked) rate. This causes the lengths of the muscle spindles within the quadriceps to decrease at a normal rate, thereby decreasing the tension in the muscle spindles at a normal rate. At this stage, when the leg, muscles and spindles are all moving within normal bounds, voluntary control of the leg kicks in, to ensure that the rate of deceleration of the leg is adequate to stop the knee at a safe angle and prevent injury. In this study, we will look at whether the coupling of taus of angular action gaps ($\tau_{\alpha }$) and the Cartesian action gap of the leg ($\tau_{d}$) are controlled using $\tau_{g}$ during this type of patellar reflexive movement (see Fig. 1a).

Although the mathematical formula for the taug equation is derived from a bouncing ball movement in the presence of gravity [7], it is worth noting that the electromyographical activity of the muscles causing the involuntary patellar reflex is very short (about 40 ms) compared with the whole movement duration [22, 23]. So after the offset of muscle activation, the remainder of the movement produced by the initial reflex could be partially regulated by gravity. To control for the possible effects of gravity on the movement of the limb, we decided to elicit the patellar reflexes in two different planes of motion: (1) in the typical sitting position with the lower limb positioned vertically with respect to the floor so that the influence of the gravitation field would be present, and (2) in a lying side position where the lower limb was positioned horizontally with respect to the floor thereby minimizing the influence of gravity.

## Methods and materials/instruments

### Participants and task

Six participants (four males and two females; mean age: mean ± standard deviation (std) = 24.2 ± 2.5 years old; mean height: 175.5 ± 8.5 m; mean weight: 74.2 ± 18.3 kg) participated in this study. The study was approved by the local ethics committee of University of Isfahan (Isfahan, Iran; IR.UI.REC.1397.101). The participants had not taken part in any similar experiment and were unaware of the aims of the experiment. The participants gave written informed consent and understood that they were free to leave at any time. The participants did not suffer from any motor or neuromuscular disease and had no history of any neurological disorder.

### Procedure

#### Vertical plane

To produce the patellar reflex movement, participants were asked to sit in a chair that was sufficiently high so that their legs were able to hang freely over the edge when they were seated [22]. An appropriate and medically approved reflex hammer was used to evoke the reflex. The hammer was attached to a pendular structure that could freely swing when held at the end. To hit the correct spot at the right speed, it was released at a 90° angle without applying any extra force. In order to maximize the chances of successfully triggering a reflex with a large magnitude, the best spot on the participant’s knee for tapping was chosen through trial and error [24]. In addition, to maximize the jerk and muscle contraction when eliciting the knee jerk, the Jendrassik manoeuvre was used [25]. To this end, participants were instructed to interlock their left and right hands and pull in response to an oral prompt given before the tendon was tapped. About two seconds after this manoeuvre began, the participant’s patellar tendon was tapped, and the reflex was recorded. For each participant, the reflex test was performed 10 times with a 30 s interval between each reflex.

#### Horizontal plane

**Fig. 2:**
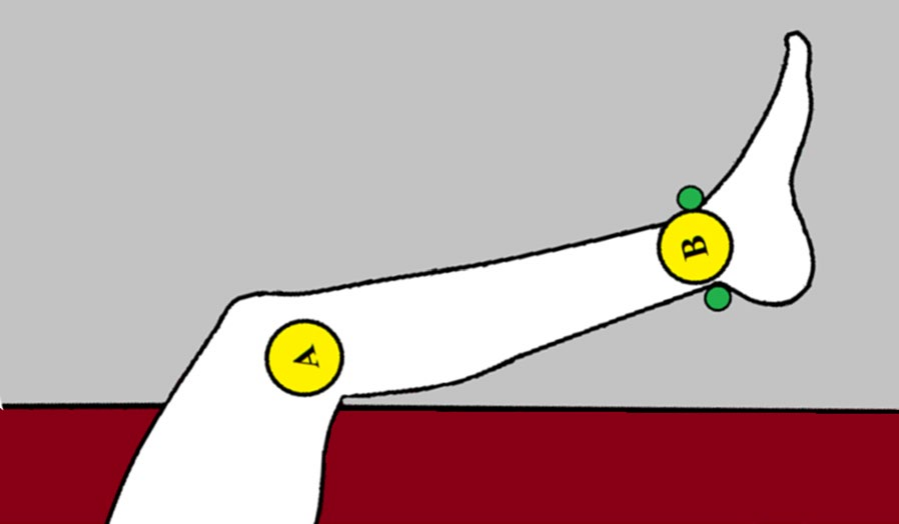
The top view of participant’s positioning in horizontal condition. Markers A and B are placed as the vertical condition. The green spots indicate the places where the rope was supporting the leg

Participants were asked to lie down in a comfortable position on the right-hand side of the body on a standard mattress (see Fig. 2). In order to ensure the body position was as similar to the one described above in the sitting position, the resting leg, that was laying on the mattress, was positioned in such a way that the thigh was at a 90° angle to the upper body. A 2.8 m rope attached to the ceiling was loosely wrapped around the ankle so that was still free to move in the horizontal plane. For each participant, the reflex test was performed 10 times with a 30 s interval between each reflex.

### Recording

To record the patellar reflexive movement, two circular markers were attached to the participant’s knee and ankle (Fig. 1a). A 120 frame per second camera recorded the markers’ location. The camera was positioned 80 cm from the participant’s leg and parallel to the two markers (see Fig. 1a). The position of the centre of each of the two markers was extracted 120 times a second through a post-processing procedure available in MATLAB software (MathWorks Inc.). The position of marker A was considered as the origin of the Cartesian coordinates.

The raw data were filtered using a second-order Butterworth low pass 8 Hz filter type 1 and the movement action gaps were obtained using the following Eq. (1):

1 $$X_{AG} \left( t \right) = x\left( t \right) - x_{end} ,Y_{AG} \left( t \right) = y\left( t \right) - y_{end}$$

where *x* and *y* represent the position of the ankle in Cartesian coordinates over time, where *x*_*end*_ and *y*_*end*_ represent the position of the ankle at the end of movement, and *X*_*AG*_ and *Y*_*AG*_ represent the action gaps of the *x* and *y* coordinates.

Ten representative action gaps for one individual are shown in Fig. 1b. The angle of the leg *β* (see Fig. 1a) was obtained by calculating the angle between the vertical coordinate and the line that connects the centres of the two markers. The angular action gap (*α*) was calculated as:

2 $$\alpha \left( t \right) = \beta \left( t \right) - \beta_{end}$$

### Analysis of the behavioral data

The mean and standard deviations of the amplitude, peak velocity and time to reach peak velocity of the ankle displacement and angular movement for all participants for vertical and horizontal conditions are reported in Table 1. All the variables were not significantly different in the Vertical and Horizontal conditions (paired sample *t*-test; for all comparisons: p > 0.324).

**Table 1:** The amplitude, peak velocity, and time to reach peak velocity for both ankle displacement and angular movement for all participants

Condition	Ankle displacement		Angular movement			
	Amplitude (cm)	Peak velocity (cm/s)	Time to peak velocity (ms)	Amplitude (rad)	Peak velocity (rad/s)	Time to peak (ms)
Vertical	8.8 (± 3.2)	39.1 (± 17)	158.3 (± 18)	16.5 (± 5.1)	73.8 (± 31)	160.2 (± 19)
Horizontal	7.8 (± 1.2)	46.9 (± 15)	158.1 (± 20)	15.1 (± 2.7)	89.7 (± 31)	158.1 (± 20)

Standard deviations are in brackets

From the displacement data the ankle movement action gaps (*d*_*AG*_) were calculated using Eq. (3).

3 $$d_{AG} \left( t \right) = \sqrt {X_{AG}^{2} \left( t \right) + Y_{AG}^{2} \left( t \right)}$$

The tau of the ankle displacement ($\tau_{d}$) and the tau of the angular movement ($\tau_{\alpha }$) were calculated using Eqs. (4) and (5).

4 $$\tau_{d} = d_{AG} \left( t \right)/\dot{d}_{AG} \left( t \right)$$

5 $$\tau_{\alpha } = \alpha \left( t \right)/\dot{\alpha }\left( t \right)$$

In keeping with other studies, only the part of the movement in each trial that was greater than 10% of the trial’s peak velocity was analysed [12].

Using the time series in which the movement was generated, $\tau_{g}$ was calculated using Eq. (6)

6 $$\tau_{g} = 0.5\left( {t - T^{2} /t} \right)$$

where, *T* is the duration of the movement and *t* is time as the movement evolves.

**Fig. 3:**
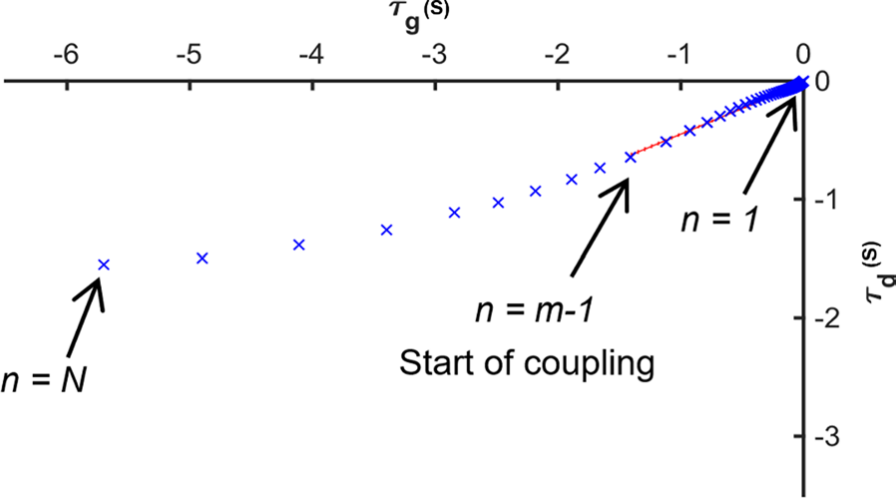
τ_g_ plotted against τ_d_ for one trial. The movement starts at sample point n = N and ends at sample point n = 1. The red line is the regression line fitted to samples from the start of coupling (n = m − 1) to end of the movement

After calculating the different taus,$\tau_{d}$, $\tau_{\alpha }$ and $\tau_{g}$ for each trial, the strength and duration of the coupling between the relative taus ($\tau_{d}$–$\tau_{g}$ and $\tau_{\alpha }$–$\tau_{g}$) was calculated in the following way [12]. When $\tau_{g}$ is plotted against $\tau_{d}$ the total number of samples equals *N* (Fig. 3; where the start of the movement is equal to *n* = *N* and the end is where *n* = 1. The first step is to fit a line to the last *m* = 10 data points (*n* = 1:*m*). The strength ($r^{2}$) of the linear regression ($r_{10}^{2}$) for those 10 points along with the standard deviation ($S10$) was then calculated. Provided that $r_{10}^{2} > 0.97$, the sample number (*m*) was increased by one sample (*m* = 11) at a time and a new regression line was fitted to samples *n* = 1:*m.* To calculate the straightness of the raw data, the parameter $d_{m} = Sm/S10$ was also calculated; where *Sm* is the standard deviation of the *m* points. By increasing *m*, if *d*_*m*_ reaches four, the samples *n* = 1:*m* − 1 were considered as the part of movement where coupling occurred. The percentage of movement (*MP*) where the linear coupling was high (> 0.97) was calculated using the equation (Eq. 7).

7 $$MP = \frac{m - 1}{N}*100$$

After analysis of the MP for the $\tau_{d}$–$\tau_{g}$ and $\tau_{\alpha }$–$\tau_{g}$ couplings in both conditions, the data were then analysed by defining a 2 (Plane: vertical and horizontal) × 2 (Coupling type: $\tau_{d}$–$\tau_{g}$ or $\tau_{\alpha }$–$\tau_{g}$) repeated measures ANOVA (rmANOVA) to study the effect of each plane on the MP of coupling between the different action gaps and taug. Bonferroni corrections were applied where necessary and the significance level as set at p < 0.05.

## Results

**Fig. 4:**
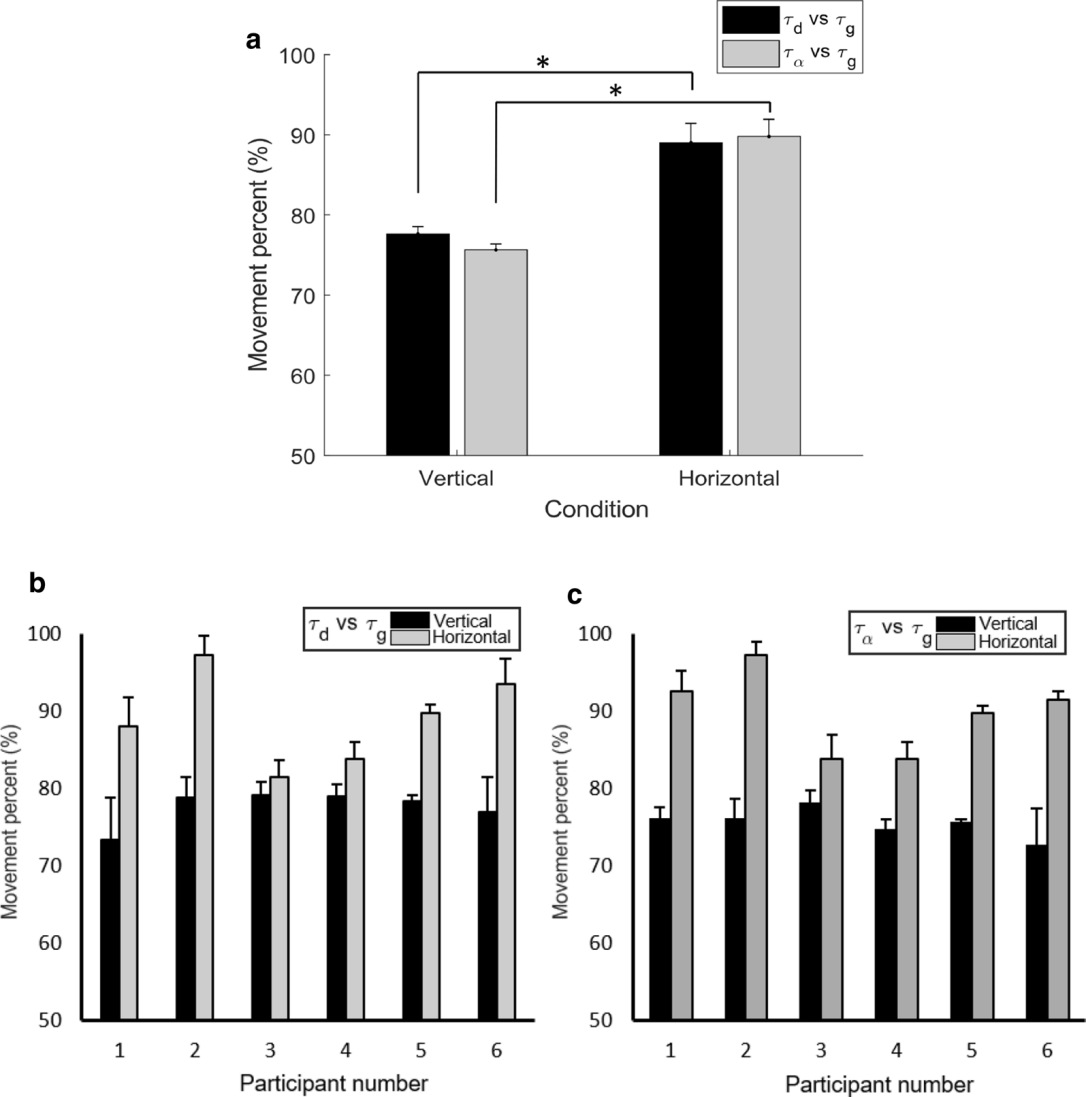
Movement percentages (MP) for the different coupling types in both the vertical and horizontal conditions. **a** shows the results averaged across all participants, while **b** and **c** show the data for individual participants. The columns represent the mean MP values for couplings for the $\tau_{d}$ and $\tau_{g}$ and $\tau_{\alpha }$ and $\tau_{g}$ gaps. The error bars represent the standard error and asterisks show the significant differences. Note how there is a significant difference between the horizontal and vertical conditions for both types of coupling

The rmANOVA results revealed a significant main effect of Plane (Fig. 4; F_(1,5)_ = 25.8, p = 0.004, η_p_^2^ = 0.838), with higher values of MP being found in the Horizontal plane (89.4% ± 2.2%) compared to the Vertical plane (76.6% ± 0.6%). On the other hand, the difference in the Coupling type was not significant (p = 0.573). Furthermore, the results showed a significant Plane × Coupling type interaction (F_(1,5)_ = 63.45, p = 0.001, η_p_^2^ = 0.927), with post-hoc comparisons revealing higher MP values in the horizontal plane for both displacement (89.0% ± 2.4%) and angular (89.8% ± 2.1%) action gaps compared to the vertical plane (displacement:77.6% ± 0.9%; angular: 75.6% ± 0.7%; for both comparisons: p < 008). This was true for all participants (see Fig. 4).

## Discussion and conclusion

According to general tau theory, voluntary self-paced movements, such as reaching for a static object, are guided by coupling the tau of the action gap with a tau guide ($\tau_{g} )$ generated intrinsically by the central nervous system [4]. It is known from the literature, that the production and guidance of reflexive movements are very different from voluntary movements. Reflexive movements are classed as innate, simple, ballistic patterns of movement that originate in the spinal cord or in the brain [18].

The main aim of this study was to see if the control of the trajectory of a movement that originates from a reflex can also be explained by general tau theory. To this end, this study looked at a simple spinal reflex, the patellar reflex, which was initiated under two different conditions (horizontal and vertical) to control for the possible influence of gravity on limb movement. In the vertical condition the potential influence of gravity was high, whilst in the horizontal condition the influence of gravity was seen as being negligible. Having the two different conditions allowed us to determine if the amount of movement coupling was due to acceleration under the influence of gravity or some kind of prospective control that ensues after a reflexive movement is initiated to prevent injury and protect the limb [26].

For the vertical condition where the subject sat in a chair with the limb positioned vertically with respect to the floor, the results showed that both $\tau_{\alpha }$ and $\tau_{d}$ were tightly coupled to an intrinsic tau guide $\tau_{g}$ (r^2^ > 0.97) for over 75% of the movement (MP > 75%). A possible interpretation of this high degree of coupling and percentage of movement tau-coupled (MP) could be due to the mathematics for taug (derived from the form of movement of a bouncing ball) being confounded by the effects of gravity and not be due to any type of prospective control as hypothesised by general tau theory.

To counteract this, the second horizontal condition was introduced to control for the effects of gravity and to confirm that a strong coupling, and percentage of movement that is tau-coupled (MP), still existed when any effects of gravity were minimised. In this case the control of the reflexive movement showed that the $\tau_{\alpha }$ and $\tau_{d}$ couplings to the intrinsic $\tau_{g}$ were equally as high as those found in the vertical condition. More importantly, the percentage of the tau-coupled movement was significantly greater in the horizontal plane (MP = 89.4% ± 2.2%) compared to vertical plane (MP = 76.6% ± 0.6%; p < 0.05). Both results reinforce the idea that the temporal control of movement when a patellar reflex is initiated, is intrinsically guided in a way that is similar to voluntary movements.

Our results suggest that even though voluntary and involuntary movements are generated in different levels of the brain and spinal cord, they are both still coupled to a more universal taug neural imprint that is adapted to gravity. Taking this into consideration, one could suggest that the scope of tau theory could be extended so it becomes a more general theory for describing the neural control of both voluntary and involuntary movements. Secondly, given that the patellar reflex is present from infancy, one can conclude that there are innate patterns of neural activity that correlate with taug to help plan and guide movement. Most probably, the innate patterns of neural activity were gradually generated during evolution through the interaction of the organism with gravity, one of earth’s most important environmental factors.

To conclude, general tau theory hypothesizes that the intrinsic pattern generated inside the nervous system guides the control of motor output. In this study we were interested in measuring and modelling the motor output observed during a patellar reflexive movement. Future work should look to extend this research and attempt to capture the patterning of information that takes place inside the brain as the control of a reflexive movement unfolds.

## Data Availability

The datasets used and/or analyzed during the current study are available from the corresponding author on reasonable request.
